# Collision Cross-Section Calibration Strategy for Lipid Measurements in SLIM-Based High-Resolution Ion Mobility

**DOI:** 10.1021/jasms.2c00067

**Published:** 2022-06-02

**Authors:** Bailey S. Rose, Jody C. May, Allison R. Reardon, John A. McLean

**Affiliations:** Center for Innovative Technology, Department of Chemistry, Vanderbilt Institute of Chemical Biology, Vanderbilt Institute for Integrative Biosystems Research and Education, Vanderbilt-Ingram Cancer Center, Vanderbilt University, Nashville, Tennessee 37235, United States

## Abstract

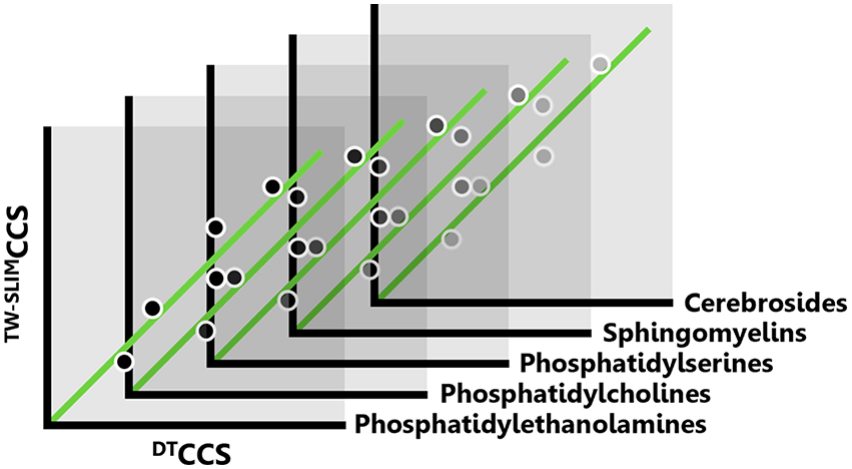

Structures for lossless ion manipulation-based high-resolution ion mobility (HRIM) interfaced with mass spectrometry has emerged as a powerful tool for the separation and analysis of many isomeric systems. IM-derived collision cross section (CCS) is increasingly used as a molecular descriptor for structural analysis and feature annotation, but there are few studies on the calibration of CCS from HRIM measurements. Here, we examine the accuracy, reproducibility, and practical applicability of CCS calibration strategies for a broad range of lipid subclasses and develop a straightforward and generalizable framework for obtaining high-resolution CCS values. We explore the utility of using structurally similar custom calibrant sets as well as lipid subclass-specific empirically derived correction factors. While the lipid calibrant sets lowered overall bias of reference CCS values from ∼2–3% to ∼0.5%, application of the subclass-specific correction to values calibrated with a broadly available general calibrant set resulted in biases <0.4%. Using this method, we generated a high-resolution CCS database containing over 90 lipid values with HRIM. To test the applicability of this method to a broader class range typical of lipidomics experiments, a standard lipid mix was analyzed. The results highlight the importance of both class and arrival time range when correcting or scaling CCS values and provide guidance for implementation of the method for more general applications.

Ion mobility interfaced with mass spectrometry (IM-MS) has become an important technique for the analysis of complex biological samples.^1−6^ Similar to chromatography, the added structurally selective separation capabilities of IM improve analyte coverage and aid in the discrimination of isomeric and isobaric species that otherwise hinder comprehensive analysis when using MS alone.^7,8^ Although chromatographic methods can be extensively optimized to enhance chemical selectivity and chromatographic resolution, achieving reproducible retention times across different laboratories remains a challenge.^9−11^ Conversely, there are fewer options to tailor the selectivity in IM as separations are based directly on an intrinsic physical property of the analyte, namely its gas-phase structure or structures. Because of this, IM-derived collision cross section (CCS) values add a highly reproducible metric for filtering and annotating features derived from mass spectra while simultaneously providing a specific structural descriptor for molecular species.^12−14^

Collision cross section values can be directly derived from classical electrodynamics using uniform-field drift tube ion mobility (DTIM) measurements via the fundamental low-field Mason–Schamp equation (^DT^CCS).^15,16^ On the other hand, traveling wave ion mobility (TWIM)-based techniques use dynamic electric fields for separation, so CCS determination is less straightforward. Though progress has been made toward derivation of a functional first-principles equation from traveling wave fundamentals,^17^ in practice, traveling wave collision cross section values (^TW^CCS) are typically obtained through calibration from experimentally measured arrival times of calibrants with known or agreed upon ^DT^CCS values.^18−22^ Calibrating ^TW^CCS introduces error in the form of a bias from fundamentally measured reference values (typically DTIM) that is dependent on both the functional equation form used in the calibration as well as the structural similarity of the calibrants and analytes.^20,23−27^ However, with appropriate considerations, these calibration methods can regularly achieve biases of <2% and have shown high interlaboratory reproducibility (<1%).^13,28−30^ This has led to widespread adoption of CCS as a compound annotation parameter and the construction of many CCS libraries to support such studies.^13,28,31−35^

While the conventional resolution range of commercially available DTIM and TWIM systems (40 to 60)^7^ has been successful in discriminating many molecular species in complex spectra, there remain many more structurally similar isomeric and isobaric species that require higher resolution.^36,37^ Despite the importance of DTIM in providing direct CCS measurements from first-principles theory, DTIM instruments rarely achieve resolving powers above ca. 100. Recent advances in structures for lossless ion manipulations (SLIM) technology have enabled the development of a TW-based high resolution IM (HRIM) system with resolving powers in excess of ∼200.^38−40^ The increased ion mobility resolution of SLIM-based HRIM has enabled the separation of many biologically relevant compounds of various chemical classes as well as the elucidation of previously unseen molecular features.^41−44^ To support the proper annotation of these additional features, robust calibration methods need to be developed and validated such that SLIM-based CCS values (^TW-SLIM^CCS) can be reliably and reproducibly derived from HRIM measurements.

Calibration of CCS for TW-SLIM has been explored previously in a limited capacity using variously modified TWIM calibration protocols. Whereas fundamental differences between the TWIM platforms used in these studies raised concerns regarding the influence of ion heating, the results provided evidence that calibrated CCS values are not significantly influenced by TW separation parameters such as wave height and amplitude, and CCS biases under 2% were achievable in most cases.^40,45,46^ Prior investigations into the utility of conventional resolution CCS databases have demonstrated the importance of low bias and high precision (reproducibility and repeatability) for untargeted applications, as lowering database search tolerances below 1% can drastically reduce the number of candidate identifications and improve annotation confidence.^47^ With the inherently higher precision of HRIM this is even more salient, as many biologically relevant isomers exhibit CCS differences of less than 2%.^37^ Therefore, ^TW-SLIM^CCS calibration with expected biases under 1% and high reproducibility (<0.5% RSD) could provide improved confidence in the expansion of database matching using HRIM.

Here, we describe the development and evaluation of a simple, reproducible ^TW-SLIM^CCS calibration strategy focused on lipids. Lipids are a functionally and structurally diverse class of biomolecules with a high prevalence of isomers and distinct CCS trends that can aid in their annotation and characterization.^48,49^ Additionally, the various subclasses of lipids occupy a well-defined range of *m*/*z* and CCS values (Figure 1), which allows the parameters of the calibration approach to be confined while enabling the generation of class-specific recommendations for calibrant selection and calibration methodology. To assess the choice of calibrants, we compare the calibrated CCS obtained from structurally similar calibrant sets as well as a more broadly applicable calibrant mixture of current and widespread use. We also examine the utility of a generalizable calibration method combined with subclass-specific CCS correction factors to provide the most precise and accurate CCS determinations from high-resolution TW-SLIM measurements.

**Figure 1:**
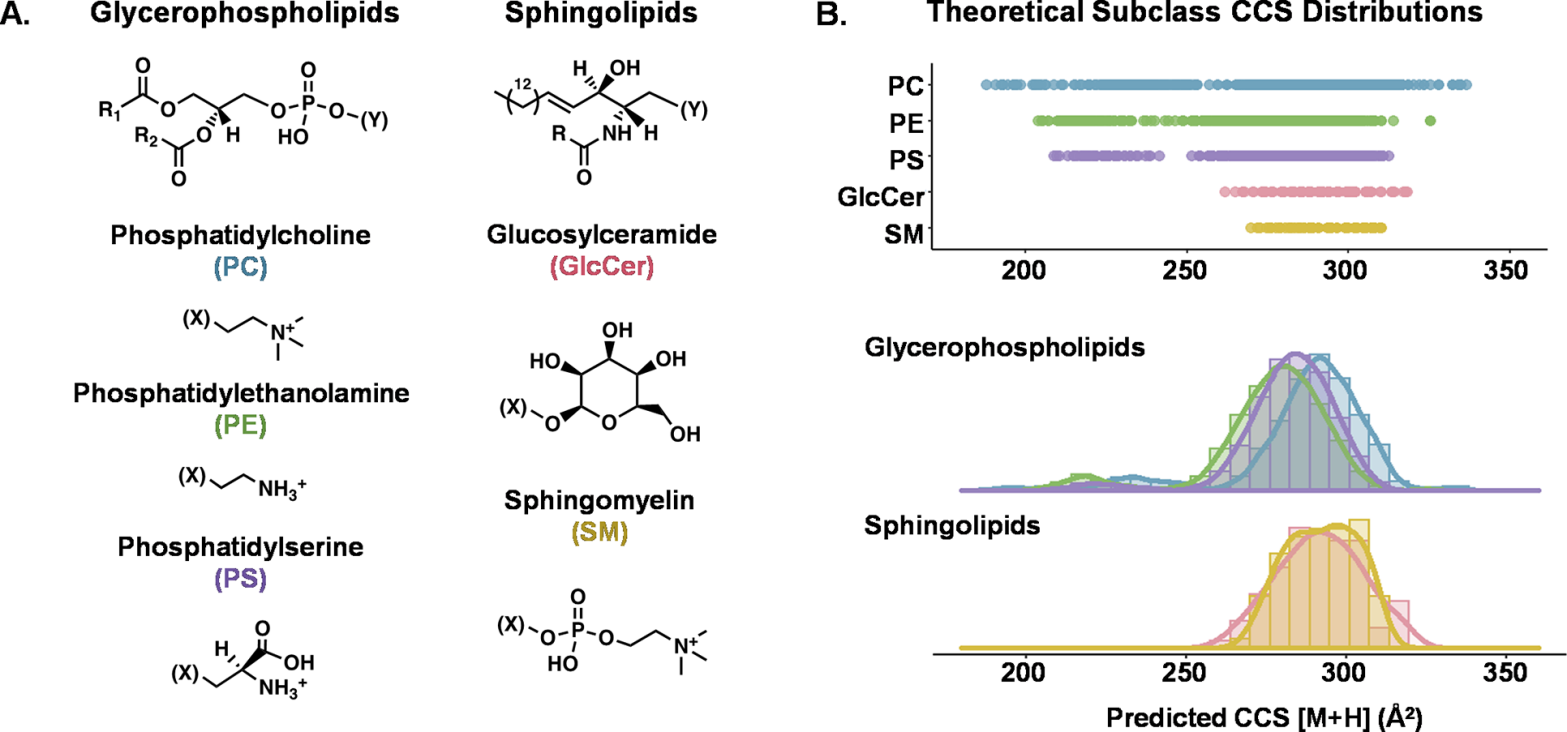
Overview of the two lipid classes and five subclasses surveyed in this study. (A) General chemical structure of each class and headgroup of each subclass. (B) Range and distribution of predicted [M + H] CCS values for all lipids from each subclass as represented in the LIPID MAPS Structural Database and predicted using LipidCCS and DeepCCS.^59,60^

## Experimental Methods

### Materials and Solvents

High-purity (Optima grade) solvents including water, methanol, acetonitrile, chloroform, and formic acid were obtained from Fisher Scientific (Hampton, NH). A tuning mixture containing betaine and a series of symmetrically branched hexakis(fluoroalkoxy)phosphazines (HFAPs, ESI-L low concentration tuning mixture, Agilent) was used for instrument tuning and CCS calibration. Purified TLC fractions of total lipid extracts including phosphatidylcholine (PC, chicken egg), phosphatidylethanolamine (PE, chicken egg), phosphatidylserine (PS, porcine brain), cerebroside (GlcCer, porcine brain), and sphingomyelin (SM, porcine brain) were purchased as lyophilized solids from Avanti Polar Lipids (Birmingham, AL) and were reconstituted in chloroform and then prepared to a final concentration of 10 μg/mL in 1:2 chloroform/methanol for analysis. A deuterated lipid standard mix of varying lipid concentrations (SPLASH, Avanti) was diluted 1:10 in 1:2 chloroform/methanol for analysis.

### Instrumentation

Data were acquired using a 13.115 m serpentine path SLIM-based HRIM platform (beta prototype, MOBILion Systems) integrated with a time-of-flight mass spectrometer (6546, Agilent Technologies), as described previously.^40,50^ Samples were introduced using a liquid chromatography system (1290, Agilent) and ionized by electrospray ionization (Jet Stream, Agilent). TW-SLIM ion mobility experiments were conducted in pure nitrogen drift gas, resulting in nitrogen-specific cross section measurements (CCS_N2_). Measurements for the standard lipid mix were also made using a commercial DTIM-MS system (6560, Agilent) for reference CCS comparison of these lipids.^51,52^

### Data Acquisition

Lipid extract samples were injected using a 3 min automated flow injection acquisition with a constant flow solvent of 0.1% formic acid in 1:1 methanol/water at a carrier flow rate of 70 μL/min and an injection volume of 10 μL. Reversed-phase liquid chromatography (RPLC) was used for the standard lipid mix, and detailed parameters, solvents, and gradients can be found in Figure S1. In all cases, the ESI source was operated in positive-ion mode using the following conditions: nebulizer pressure, 20 psi; sheath gas flow rate, 12 L/min; sheath gas temperature, 275 °C; drying gas flow rate, 5 L/min; drying gas temperature, 325 °C; capillary voltage, 4000 V; entrance nozzle voltage, 2000 V. The SLIM boards were operated at ca. 2.500 Torr. TW-based separation was performed using a wave speed of 180 m/s and a peak-to-peak wave amplitude of 40 V_pp_. These separation parameters were chosen based on optimal conditions for high resolving power in the *m*/*z* range of the lipid analytes as described previously.^40^ Data was acquired via MassHunter Acquisition (v. 9.0, Agilent) and EyeOn software (v. 0.3.0.15, MOBILion Systems). For CCS calibration with HFAPs, data for the tune mix was acquired in a separate experiment using identical instrument parameters (i.e., external CCS calibration). For calibration using the lipids, lipids observed within the spectra from each extract was selected as calibrants (i.e., internal CCS calibration). For data acquired on the DTIMS instrument, the LC and ESI source conditions were identical to those used on the TW-SLIM system. The drift tube was operated under 3.95 Torr nitrogen gas, and additional drift tube parameters were as follows: ion trap fill time, 20 ms; ion trap release time, 300 μs; drift tube entrance, 1474 V; drift tube exit, 224 V; rear funnel entrance, 217.5 V; rear funnel RF, 150 V_pp_; rear funnel exit, 45 V (matched to the QTOF autotune setting); and IM hexapole delta, −8 V. The QTOF stage was operated in low mass range (*m*/*z* 50–1700), ion slicer operated at high sensitivity and the digitizer operated at 2 GHz extended dynamic range. Single-field CCS values were obtained using HFAP drift times as described previously.^12^

### Data Processing and Software

The PNNL preprocessor (version 3.0) was used for IM-MS file conversion and drift bin compression (2:1) for the TW-SLIM data.^53^ Lipid feature arrival times (peak centroids) were extracted manually within the MassHunter IM-MS Browser (v. 10.0.1.10039, Agilent). To increase confidence in the peak selection, only features falling within the expected lipid IM-MS correlation region were considered for extraction.^54^ CCS calibration and bias calculations were performed in Excel (Microsoft).

### Guiding Calibration Theory

Calibration of CCS from commercial TW systems is commonly performed using a set of calibrants with known ^DT^CCS values.^21^ Their experimental arrival time (*t*_A_) is plotted against the corresponding “reduced” ^DT^CCS values (CCS′), as calculated using eq 1 where *z* represents the ion charge and μ is the reduced mass of the ion-neutral pair.
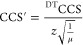
1

As described previously, conformational space occupancy arises from average density as related to the cubic volume and squared area of a given set of biomolecules, giving rise to a length squared versus length cubed relationship.^55^ Similarly, the arrival time-CCS′ relationship has been modeled using many nonlinear equation forms, but generally, power functions or polynomials are used for TW data.^18,20,21,23^ Studies using both power functions and polynomials have shown high reproducibility and relatively low biases under varying TW amplitudes and speeds.^40,45^ Trinomial equation forms resulted in the lowest biases from ^DT^CCS values in these prior studies and therefore are used as the basis for CCS calibration in this work (eq 2).
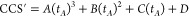
2

Calibrated CCS bias from reference ^DT^CCS values (CCS_ref_) is used as a comparison metric to estimate the accuracy for the different calibration methods (eq 3). Values from a large database of standardized ^DT^CCS values, the Unified CCS Compendium, were used as reference values for lipids with a database *m*/*z* match.^34^
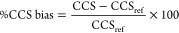
3

## Results and Discussion

Here, we evaluate three strategies for CCS calibration of lipid measurements from a SLIM-based HRIM platform: (1) The broadly available HFAP tuning mixture was used for calibration following the protocols set forth in previous TW-SLIM studies which are in turn modeled from conventional TW calibration practices; (2) calibration using subclass-specific lipid calibrant sets was evaluated to determine if chemically similar calibrants significantly impacted the ^TW-SLIM^CCS measurement bias from reference CCS values obtained from DTIM measurements; and (3) a correction factor was applied to the calibration obtained using the HFAPs to determine if a more generalizable calibration protocol could be developed with similar or better biases observed from the first two approaches.

### HFAPs as TW-SLIM Calibrants

Here, we first evaluate a widely used set of calibrants, HFAPs, for calibration of the lipid features, which is desirable from the standpoint that this tuning mixture is widely accessible and currently utilized for tuning and benchmarking the instrumentation used in this study. Additionally, the ions from this mixture cover the entire experimental arrival time range, and reference ^DT^CCS values of these compounds are available to assess CCS measurement accuracy.^12^ Using HFAPs with the calibration method described in eqs 1 and 2 yielded calibrated ^TW-SLIM^CCS values with high reproducibility (<0.35% RSD for all lipids, Figure S2); however, systematic subclass-dependent biases of +2–3% from drift tube values were observed across all five lipid subclasses (Figure S3). To assess the contribution of the uncertainty associated with the reference HFAP ^DT^CCS values (∼0.1%), simulated calibrations were performed, similar to previous studies.^12,56^ These calibrations are described in detail in the Supporting Information and resulted in CCS uncertainty of 0.04% which could be attributed to that of the reference values. A systematic bias of ∼2% is consistent with the results from Hines et al. where HFAP was used to calibrate lipids and was attributed to a structural mismatch between the calibrants (phosphazines) and analytes (lipids).^24^ A visualization of the calibrant plot resulting in this systematic bias is shown in Figure S4.

### Lipid-Specific Calibrants

**Figure 2:**
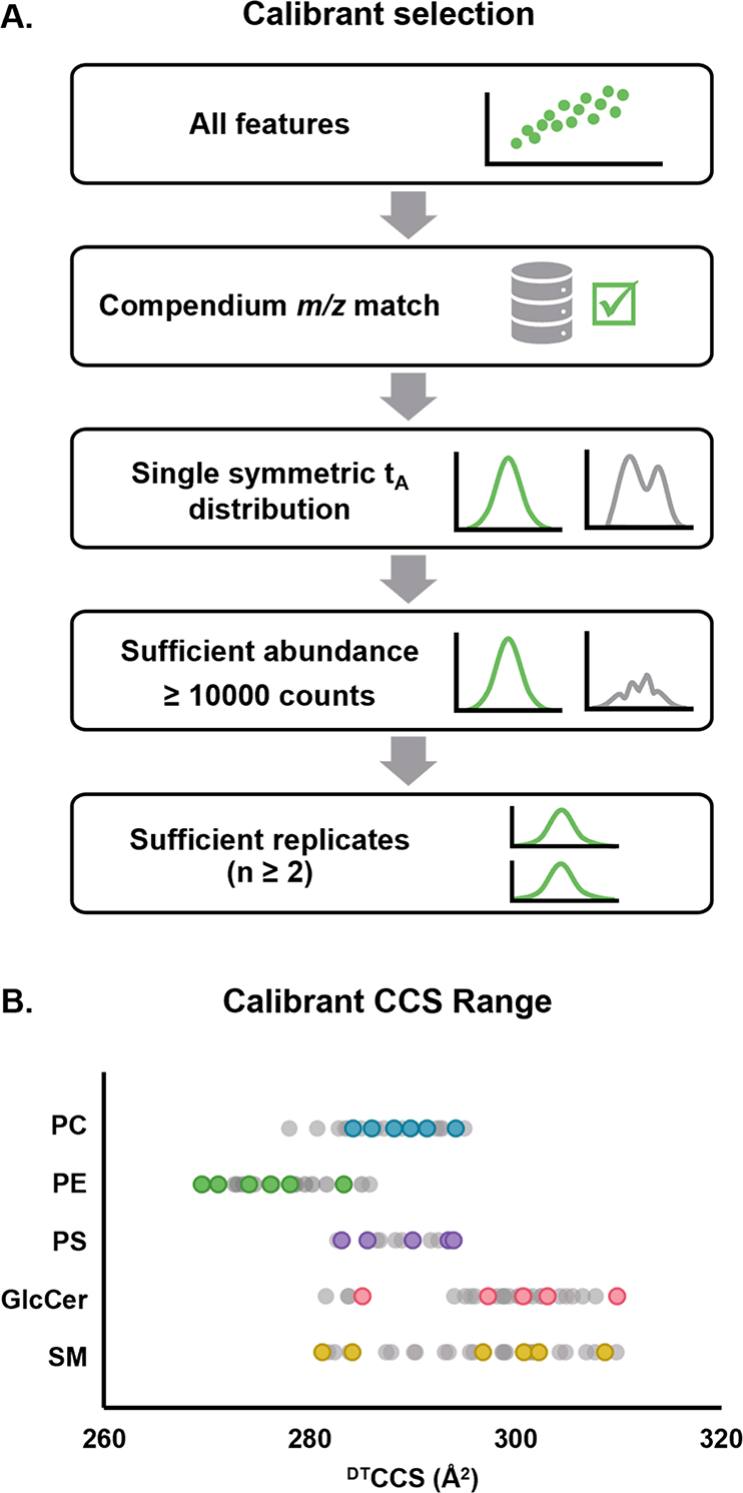
Subclass-specific calibrant selection. (A) Criteria used to select calibrant lipids from each subclass extract. (B) CCS range of each set of calibrants chosen as compared to the range of all observed lipid features (gray) in each class.

It is well-documented that the choice of calibrant plays a significant role in the resulting TWIM CCS calibration accuracy.^23−25^ As structurally similar calibrants have been shown to improve CCS bias in many cases, we next curated subclass-specific sets of lipid calibrants which are observed in each lipid extract using the reference ^DT^CCS values in the Unified CCS Compendium and criteria outlined in Figure 2a. Briefly, features were only considered as calibrants if they exhibited a single symmetrical HRIM profile with no indication of multiple contributing structures (i.e., peak asymmetry or splitting), and a ^DT^CCS value matching the feature *m*/*z* was also present in the Compendium. Additionally, quality thresholds of high abundance and multireplicate observations were used to further screen the candidate calibrants. This selection process resulted in five to seven calibrant lipids from each subclass extract, each of which spanned most of the arrival time range of the analytes (Figure 2b).

**Figure 3:**
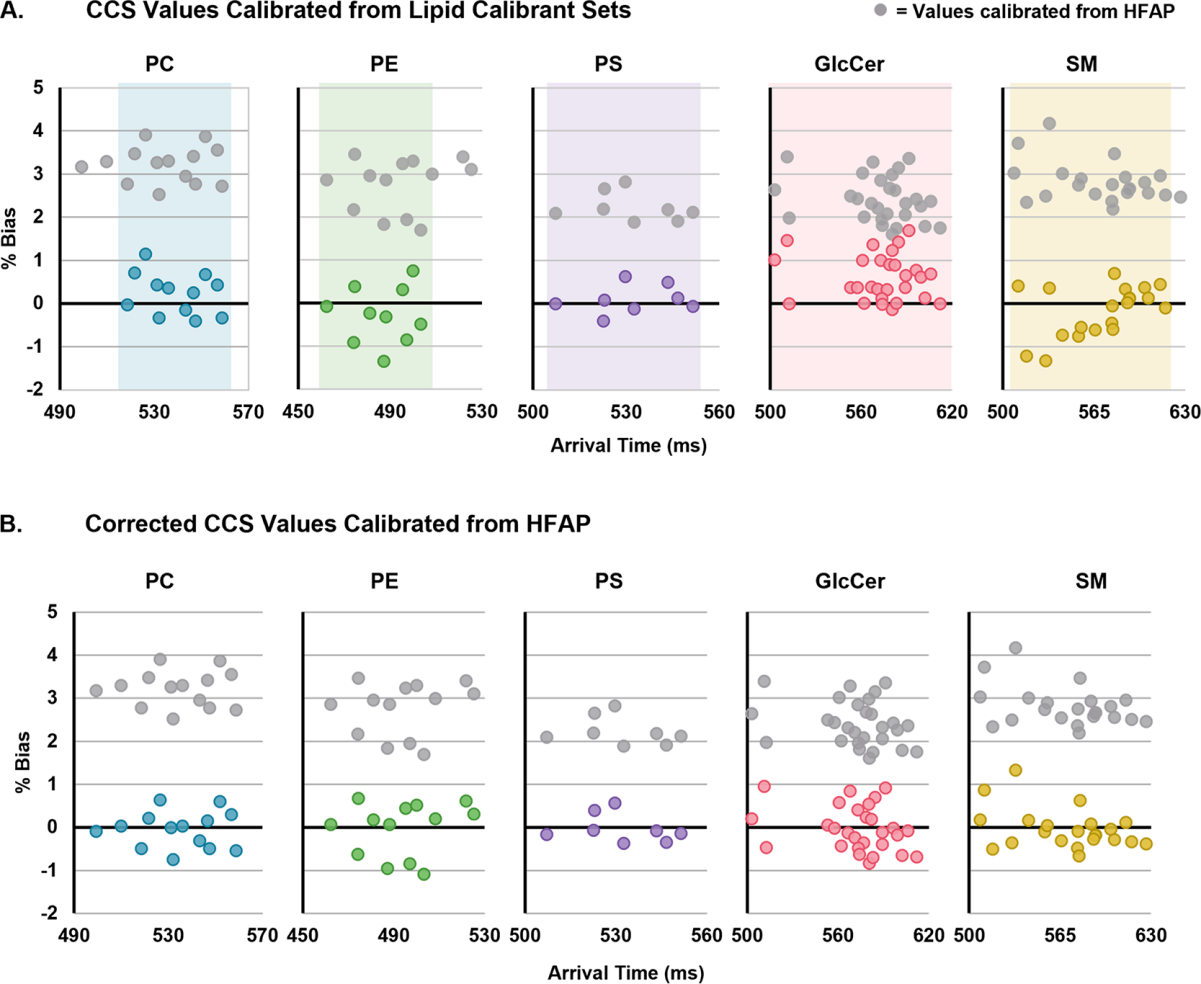
CCS biases from triplicate ^DT^CCS values of two types of calibration strategies. In both panels, gray values represent those calibrated using HFAP calibrants with no added correction factor. (A) Lipids calibrated from lipid calibrants within the same subclass. Shaded regions represent the arrival time ranges of the calibrant sets. Analyte lipids falling outside the calibrant range are excluded due to the high error associated with extrapolating polynomials. (B) Lipids calibrated from HFAP calibrants with an added subclass-specific correction factor.

Using lipids of the same subclass to calibrate the analytes was found to lower the CCS bias substantially to an absolute average bias of 0.48%. All lipid-calibrated ^TW-SLIM^CCS values, including those of the calibrants themselves, were found to be within 2% of the reference ^DT^CCS values, and 80% were within 1% bias (Figure 3a). While promising, this strategy requires at least four calibrants with known CCS values, which, depending on the analyte system under investigation, might not always be achievable due to the limited availability of appropriate analytical standards. Additionally, the potential presence of isomeric species in the lipid extracts results in some ambiguity in the structural assignment of the calibrants even when no separation is observed and may contribute to the associated calibration error. Finally, for this approach, it is important to choose calibrants which span the full range of analyte arrival times due to the high errors associated with extrapolating trinomial fits. Whereas the more commonly used power fits perform better when the calibration range must be extrapolated, these equations can result in higher CCS biases than the trinomial fit used here.^40^ Thus, a calibration procedure that incorporates calibrants spanning a broad range of arrival times with a trinomial calibration equation is desirable.

### Generalizable Calibration Using HFAPs

**Figure 4:**
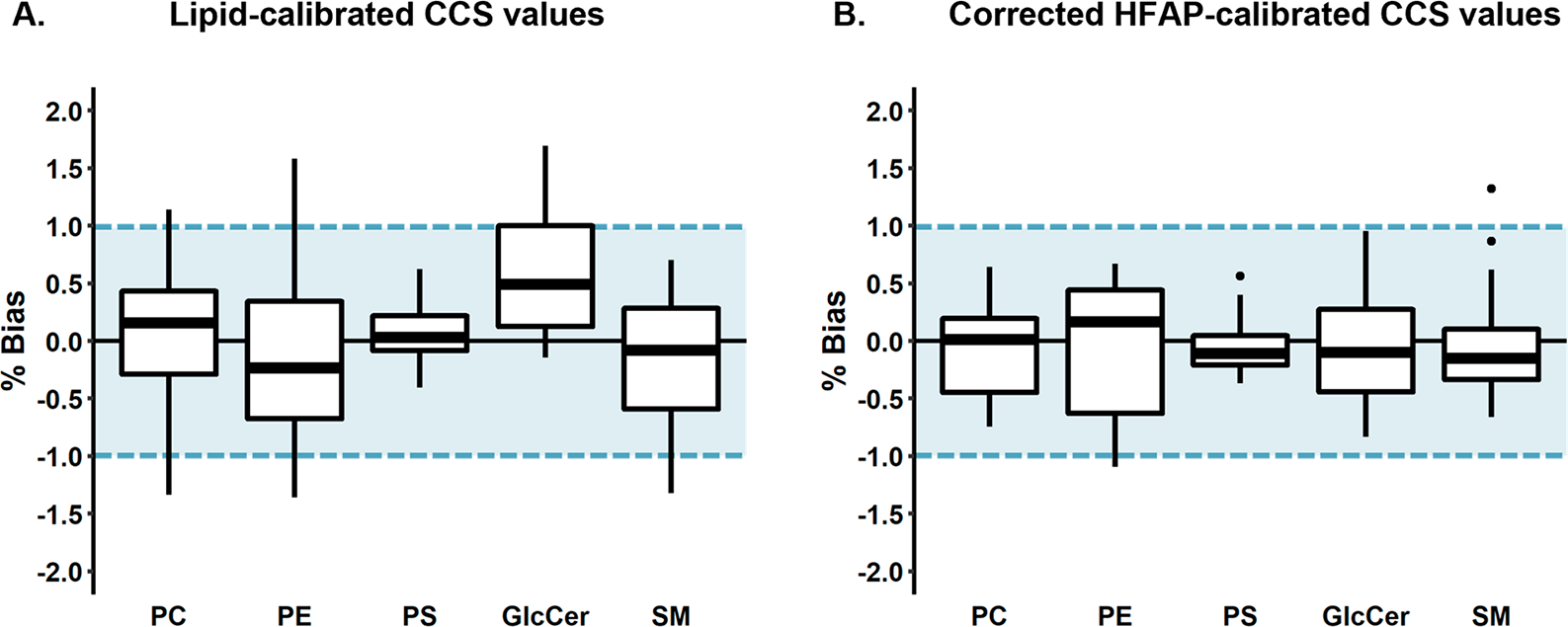
CCS bias distributions for (A) CCS values calibrated from calibrants within the same subclass and (B) values calibrated from HFAPs with an applied correction factor. Center lines represent the median of each subclass. Markers outside whiskers represent statistical outliers. Blue shaded regions represent the target bias of ±1%.

Aside from using new calibrant sets, there is precedent for adjusting calibrated CCS bias using corrections of varying degrees of complexity. Including a correction factor to the ^TW^CCS calibration has been used to address various systematic contributions to bias, including instrumental time delays, ion motion disparities, and calibrant structural differences while providing a more generalizable calibration protocol accessible to a broader community of researchers.^12,17,26,57^ Here, we apply a subclass-specific semiempirical correction to the trinomial fit based on the HFAP calibrants. To achieve this, each ^TW-SLIM^CCS determined from HFAP calibration was rescaled to the average bias of its respective subclass using a simple linear correction factor. This strategy lowered the bias to an absolute average of 0.38%, which is a lower bias than what was observed with using lipid-specific calibrants (Figure 3b). Using a subclass-specific correction factor also resulted in less bias variability than the lipid calibrants, with 98% of biases under 1% and all values under 1.5% (Figure 4). In addition to providing lower variability, using HFAPs with a correction factor is a more straightforward and broadly applicable calibration approach. HFAPs are widely available, exogenous compounds that cover a broad experimental arrival time range, and their ^DT^CCS values have been thoroughly vetted by the community. This mixture is also stable and structurally defined, whereas lipids are more prone to solution-phase degradation and may also have unresolved structural contributions to their mobility profiles. For these reasons, the correction factor applied to HFAP-based calibration is a more practical strategy for obtaining the CCS of lipids from HRIM measurements.

Using the corrected HFAP calibration, an empirical HRIM-derived database of over 90 ^TW-SLIM^CCS values was compiled (Table S2), including over 20 lipids that were previously unresolved by conventional resolution DTIM. The cerebrosides, in particular, produced many new spectral features, likely as a result of isomeric variations in the sugar headgroup. In many subclasses, new conformational trendlines were observed in the high-resolution data set that were obscured by higher abundance isobars in conventional resolution measurements. In all cases, mapping the correlations of the high-precision calibrated CCS values will be essential for the characterization of the newly resolved features. The publication of this database provides a significant resource to the community and may be applied to future studies in HRIM lipid annotation and characterization. Similar to databases for molecular annotation at varying MS resolving power, we find this is also, and potentially more, necessary for ion mobility-derived CCS values.

### Applications to Other Lipids

**Figure 5:**
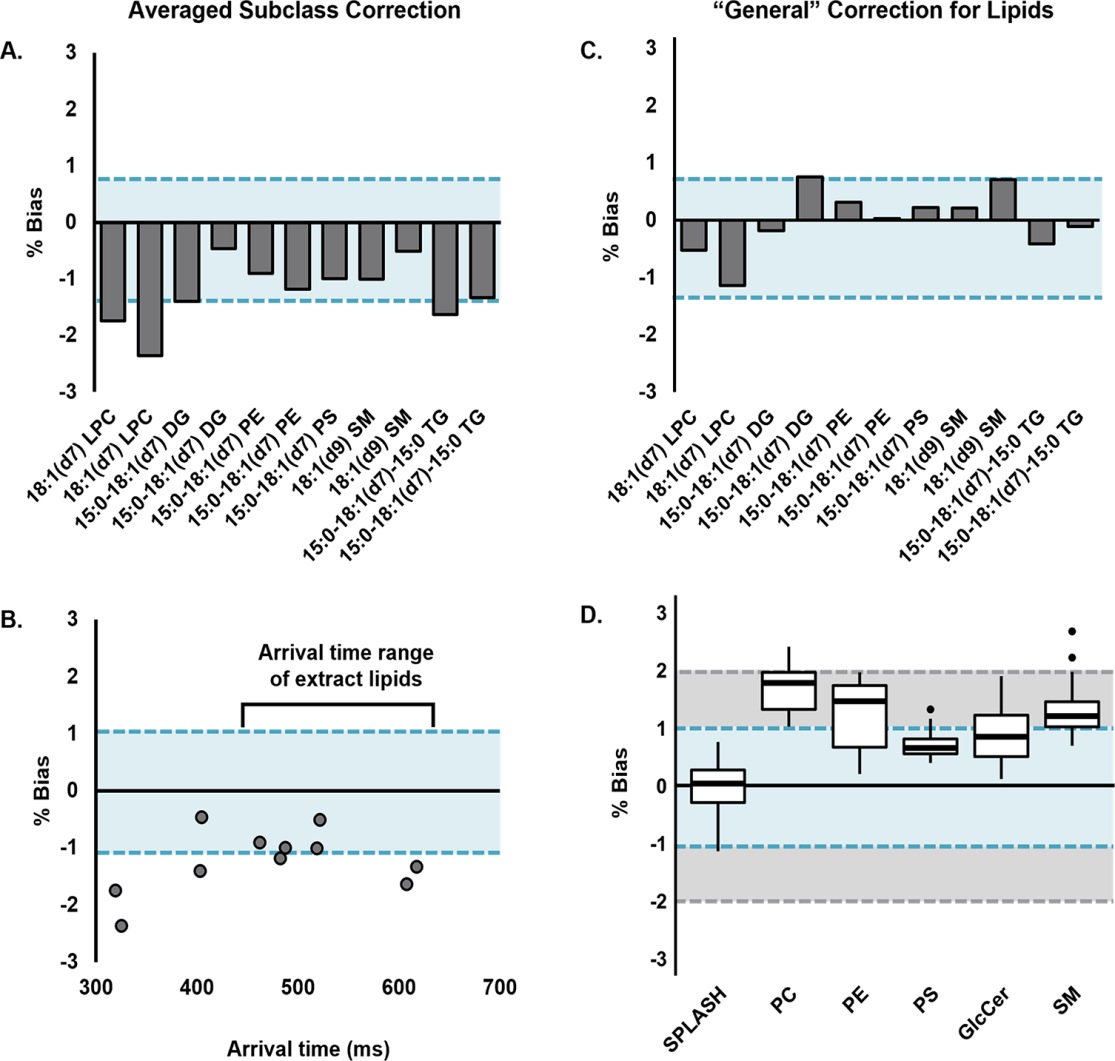
CCS biases from ^DT^CCS values of heavy-labeled lipid standard mix components. In all panels, blue shaded regions represent the target bias of ±1%. (A) Standard lipids calibrated with HFAP and an averaged correction factor derived from the five standard extracts examined in this study. Analytes are arranged in order of increasing measured arrival times. (B) Arrival time range of the standards as compared to those of the lipid extract species. (C) Standard biases when calibrated with HFAP and a “general” correction factor of 1.5%. (D) Biases of the standard mix (SPLASH) as well as lipid extract species when calibrated using the general correction factor. The gray shaded region represents the expected bias of conventional TWIMS, ± 2%.

To test the generalizability of the correction approach to broader lipidomic applications, a standard mix of heavy-labeled lipids from various classes was analyzed. For comparison, ^DT^CCS values for this standard lipid mix were measured and have been published to the Unified CCS Compendium.^58^ Because of the varied concentrations of the components and contributions from ion suppression, a reproducible signal for the largest number of standards was achieved only when analyzed with LC, which limits its utility as a calibrant. The observed features were extracted, identified, and then subjected to calibration using HFAP and an averaged correction factor of 2.7% from the extract lipids that were originally evaluated in this study. This approach overcorrected the CCS and resulted in negative biases ranging from 0.5 to 2.4, as shown in Figure 5a. The standard lipids of subclasses from which the correction factor was derived (i.e., PE, PS, and SM) had biases within the range of their corresponding extract lipid subclasses (−1.1 to 1.3%, Figure 3b), but the glycerolipids (DG and TG) exhibited higher biases in general (up to −1.6%). In addition to lipid class, arrival time range also contributed to these biases (Figure 5b). Although lysophosphatidylcholine (LPC) is within the classes examined in this study, its arrival time falls below the range of those lipids, and accordingly, the observed bias was higher (2.4%). These results suggest a smaller magnitude correction may be useful for general use in lipidomics experiments where subclass information is unknown.

While the average subclass specific correction was 2.7%, a smaller correction of 1.5% was applied as a more “general” correction to the SPLASH mix standards as empirically determined from their average absolute bias. Applying this more conservative correction resulted in lower biases, with the highest absolute value at 1.1% (Figure 5c, Table S1). As expected, application of the smaller correction factor to the other lipid extract species resulted in higher absolute biases than their subclass-specific corrections (Figure 5d). However, most values (94%) still fell within 2% bias, which is comparable to the expected CCS bias in conventional TWIM experiments and is sufficient for many applications. While not explicitly explored here, for cases where the specific lipid subclass is known, applying a second subclass-specific correction would result in even lower biases, as observed here.

## Conclusions

In this work, we evaluated the accuracy and practical applicability of various CCS calibration strategies for five lipid subclasses on a SLIM-based TWIM platform. Using a simple trinomial calibration based on a HFAP tuning mixture resulted in subclass-dependent systematic biases of 2–3% from reference ^DT^CCS values. While curation of custom calibrant sets of lipids within each subclass lowered the bias to within 0.5% on average, using subclass specific semiempirical correction factors with the more generalizable HFAP calibration provided the lowest biases (<0.4%) and variability (98% of values under 1% bias). This HFAP-based correction strategy provides a straightforward and accessible method for obtaining highly reproducible lipid CCS values, and with empirically determined correction factors it can be generalized to other compounds. Using this calibration method, we curated a HRIM CCS database of over 90 calibrated lipid values from all five subclasses, including those of many newly resolved lipid features. The routine acquisition of high precision CCS values with bias under 1% enables the construction and application of new HRIM libraries, as well as comparison to the many existing ^DT^CCS libraries. These results coupled with the high reproducibility of these values will facilitate efforts toward interlaboratory evaluation and standardization, a crucial direction of future work. Such studies will pave the way for further characterization of newly elucidated spectral features in support of untargeted applications.

## Supporting Information Available

RPLC method details (Figure S1), calibrated CCS reproducibility (Figure S2), initial calibration results (Figure S3), Tune Mix calibration plot (Figure S4), semi-empirical correction factors (Table S1), calibrated lipid CCS values (Table S2), and calibration simulations (Tables S3–S6) (PDF)
