# Supplementary material for: Collision Cross-Section Calibration Strategy for Lipid Measurements in SLIM-Based High-Resolution Ion Mobility
Source: J Am Soc Mass Spectrom. 2022 Jun 2;33(7):1229–37. doi: 10.1021/jasms.2c00067 (PMC9516683; doi:10.1021/jasms.2c00067)
Supplement: Supplementary file 1 — js2c00067_si_001.pdf [file js2c00067_si_001.pdf]

Supporting Information

A Collision Cross Section Calibration Strategy for Lipid Measurements in SLIM-based High Resolution Ion Mobility

Bailey S. Rose, Jody C. May, Allison R. Reardon, and John A. McLean*

Center for Innovative Technology, Department of Chemistry, Vanderbilt Institute of Chemical Biology, Vanderbilt Institute for Integrative Biosystems Research and Education, Vanderbilt-Ingram Cancer Center, Vanderbilt University, Nashville, TN, 37235, United States

* Corresponding Author Email: john.a.mclean@vanderbilt.edu

SI Contents:

Figure S1. – RPLC method details…………………………………………………… S2
Figure S2. – Arrival time and calibrated CCS reproducibility……………………………… S3
Figure S3. – Initial calibration results…………………………………………………… S4
Figure S4. – Tune Mix calibration plot…………………………………………………… S5
Table S1. – Semi-empirical correction factors…………………………………………… S6
Table S2. – Calibrated lipid CCS values………………………………………………… S7
Table S3-6. – Calibration simulation for evaluation of calibrant error contribution……… S10

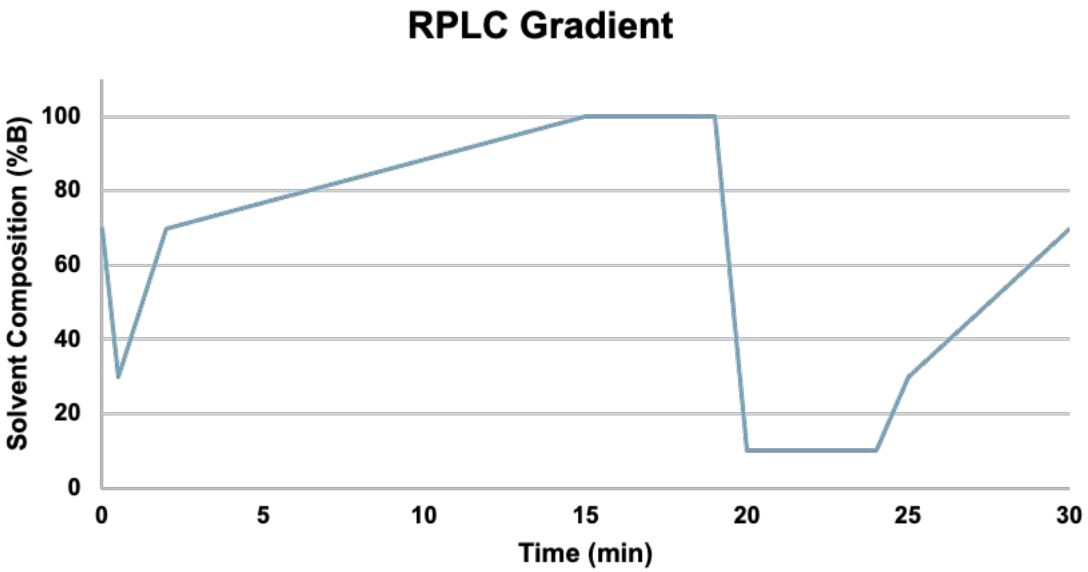

Figure S1. The standard lipid mix was analyzed using a 1290 Infinity LC system (Agilent). Reversed phase LC was performed at a flow rate of 250 µL/min using a C18 column (HypersilGold 1.9 µm, 2.1 mm x 100 mm column, Thermo Fisher) held at 40 ˚C with mobile phases consisting of 10 mM ammonium formate and 0.1% formic acid in H_2O (A) and 60:36:4 IPA:ACN:H_2O (B). The 30 minute gradient is illustrated above.

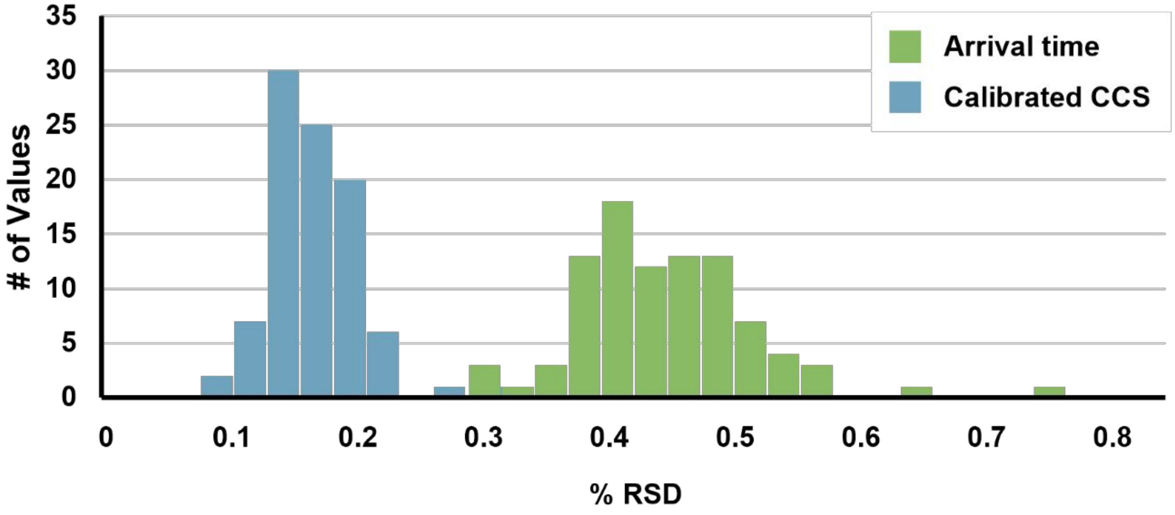

Figure S2. Inter-day reproducibility of experimental arrival times (green) and calibrated CCS values from all lipid features (n = 92).

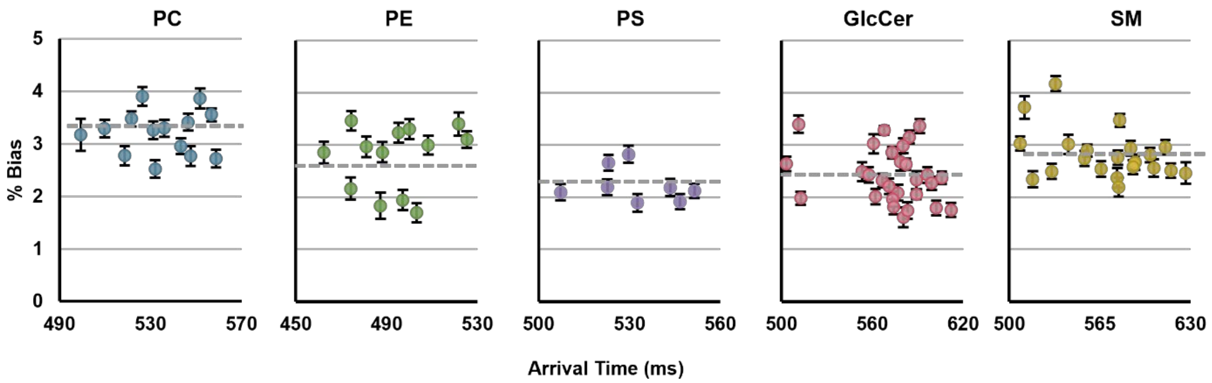

Figure S3. CCS calibration bias for all values calibrated from triplicate measurements using HFAPs with a 3rd order polynomial as compared to established ^{DT}CCS values. Dashed lines represent average biases for each subclass, and error bars represent the measurement standard deviation.

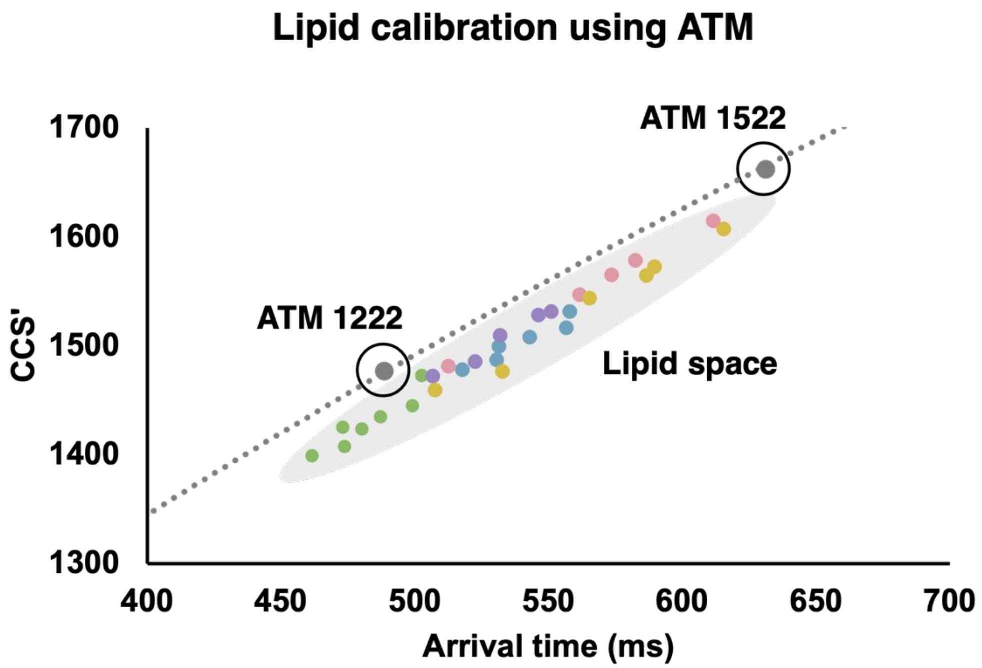

Figure S4: CCS calibration plot of the lipid analytes as compared to the Tune Mix ions *m/z* 1222 and *m/z* 1522.

Table S1. Subclass specific semi-empirically derived correction factors used for correction of HFAP-calibrated CCS values.

**Class**	**Subclass**	**Correction factor**
**Glycerophospholipids**	PC	0.0321
	PE	0.0275
	PS	0.0223
**Sphingolipids**	GlcCer	0.0241
	SM	0.0281
**SPLASH Mix**		0.0150

Table S2. Table of calibrated and corrected CCS values for all lipids using correction factors in Table S1. ^{DT}CCS values in the left column are sourced from the Unified CCS Compendium. Lipid features from each subclass that were not identified are annotated as their subclass and measured *m/z*. When arrival time features were observed for one *m/z*, peak numbers are annotated in ascending order of arrival time. Species used as calibrants in the lipid calibrant study (Figure 2) are denoted with a (*).

**Tentative ID**				**Feature description**			
**Name**	**Adduct**	**^{DT}CCS (Å^2)**	**Bias (%)**	***m/z* (measured)**	**Peak #**	**$^{TW-SLIM}$CCS (Å^2)**	**RSD (%)**
**Phosphatidylcholines**							
PC 34:01	[M+H]	283.1	0.21	760.5899	1	283.7	0.14
PC 34:01	[M+H]	283.1	0.64	760.5902	2	284.9	0.18
PC 34:01*	[M+Na]	286.0	0.00	782.5720	1	286.0	0.16
PC 34:02	[M+H]	280.6	0.03	758.5742	1	280.7	0.17
PC 34:02*	[M+Na]	284.2	-0.49	780.5566	1	282.8	0.18
PC 34:03	[M+H]	278.2	-0.09	756.5605	1	277.9	0.31
PC 36:01	[M+H]	289.4	0.15	788.6210	1	289.8	0.17
PC 36:01	[M+H]	289.4	0.60	788.6215	2	291.1	0.19
PC 36:01	[M+Na]	291.4	-0.50	810.6054	1	290.0	0.18
PC 36:01	[M+Na]	291.4	0.29	810.6051	2	292.2	0.13
PC 36:02	[M+H]	287.1	0.03	786.6056	1	287.2	0.17
PC 36:02*	[M+Na]	289.8	-0.31	808.5896	1	288.9	0.15
PC 36:03*	[M+Na]	288.2	-0.74	806.5746	1	286.1	0.17
PC 38:03*	[M+Na]	294.2	-0.55	834.6071	1	292.6	0.17
PC (*m/z* 782.57)	--	--	--	782.574233	1	293.3	0.19
PC (*m/z* 804.56)	--	--	--	804.558767	1	296.4	0.14
PC (*m/z* 832.59)	--	--	--	832.590633	1	302.9	0.17
PC (*m/z* 836.63)	--	--	--	836.624333	1	305.0	0.17
	**Average absolute % bias**		**0.33**		**Average % RSD**		**0.17**
**Phosphatidylethanolamines**							
PE 34:01*	[M+H]	271.1	0.67	718.5438	1	272.9	0.18
PE 34:01	[M+Na]	277.1	0.44	740.5270	1	278.3	0.19
PE 34:02*	[M+H]	269.5	0.06	716.5275	1	269.7	0.21
PE 34:02*	[M+Na]	274.1	0.17	738.5098	1	274.6	0.20
PE 36:01*	[M+H]	278.1	0.51	746.5752	1	279.5	0.19
PE 36:01*	[M+Na]	283.3	-1.09	768.5587	1	280.2	0.18
PE 36:01	[M+2Na-H]	284.9	0.31	790.5421	1	285.8	0.16
PE 36:02*	[M+H]	276.2	0.07	744.5589	1	276.4	0.19
PE 36:02	[M+Na]	281.0	0.20	766.5410	1	281.6	0.18
PE 36:02	[M+Na]	281.0	-0.85	766.5448	2	278.6	0.19
PE 36:03	[M+H]	274.4	-0.63	742.5467	1	272.7	0.22
PE 36:03	[M+Na]	278.7	-0.96	764.5288	1	276.0	0.25
PE (*m/z* 740.53)	--	--	--	740.5295	1	273.5	0.20
PE (*m/z* 768.56)	--	--	--	768.5599	1	285.0	0.22
	**Average absolute % bias**		**0.51**		**Average % RSD**		**0.20**

**Phosphatidylserines**							
PS 36:01*	[M+H]	285.6	0.40	790.5650	1	286.7	0.15
PS 36:02*	[M+H]	283.1	-0.16	788.5505	1	282.6	0.16
PS 38:04	[M+H]	286.7	0.56	812.5484	1	286.5	0.15
PS 38:04	[M+H]	286.7	-0.07	812.5490	2	288.3	0.16
PS 40:04*	[M+H]	293.5	-0.34	840.5801	1	292.5	0.15
PS 40:05	[M+H]	291.9	-0.08	838.5661	1	291.7	0.17
PS 40:06*	[M+H]	290.0	-0.37	836.5494	1	288.9	0.17
PS 40:06*	[M+Na]	294.0	-0.14	858.5327	1	293.6	0.14
	**Average absolute % bias**		**0.27**			**Average % RSD**	**0.16**
**Cerebrosides**							
GlcCer 36:01	[M+H-H2O]	281.0	0.20	710.6001	1	281.5	0.14
GlcCer 36:01	[M+H-H2O]	281.0	0.95	710.5992	2	283.7	0.16
GlcCer 36:01*	[M+Na]	285.1	-0.47	750.5925	1	283.8	0.12
GlcCer 40:00	[M+Na]	298.9	-0.23	808.6726	1	298.2	0.15
GlcCer 40:00	[M+Na]	298.9	0.54	808.6729	2	300.5	0.14
GlcCer 40:01	[M+H-H2O]	293.9	0.05	766.6641	1	294.0	0.18
GlcCer 40:01	[M+H-H2O]	293.9	0.58	766.6653	2	295.6	0.19
GlcCer 40:01*	[M+Na]	297.3	-0.44	806.6560	1	296.0	0.15
GlcCer 40:02	[M+H]	295.1	-0.02	782.6565	1	295.1	0.15
GlcCer 40:02	[M+H]	295.1	0.84	782.6593	2	297.6	0.08
GlcCer 42:00	[M+Na]	304.3	-0.02	836.7027	1	304.2	0.16
GlcCer 42:00 OH	[M+Na]	306.7	-0.08	852.6966	1	306.5	0.12
GlcCer 42:01	[M+H-H2O]	299.5	0.23	794.6925	1	300.2	0.17
GlcCer 42:01	[M+H-H2O]	299.5	0.70	794.6928	2	301.6	0.13
GlcCer 42:01	[M+Na]	302.9	-0.83	834.6867	1	300.4	0.19
GlcCer 42:01	[M+Na]	302.9	-0.12	834.6864	2	302.5	0.18
GlcCer 42:01 OH	[M+Na]	305.4	-0.18	850.6816	1	304.9	0.13
GlcCer 42:02	[M+H]	300.4	-0.36	810.6905	1	299.3	0.15
GlcCer 42:02	[M+H]	300.4	0.18	810.6890	2	300.9	0.11
GlcCer 42:02	[M+H]	300.4	0.92	810.6884	3	303.2	0.14
GlcCer 42:02	[M+H-H2O]	297.6	-0.13	792.6776	1	297.2	0.13
GlcCer 42:02	[M+H-H2O]	297.6	0.41	792.6778	2	298.8	0.12
GlcCer 42:02*	[M+Na]	300.7	-0.63	832.6701	1	298.8	0.15
GlcCer 42:02 OH*	[M+Na]	303.1	-0.70	848.6660	1	301.0	0.16
GlcCer 42:04 OH	[M+H]	300.2	-0.48	822.6523	1	298.8	0.14
GlcCer 43:02	[M+Na]	303.6	-0.39	846.6836	1	302.4	0.09
GlcCer 44:02	[M+Na]	307.5	-0.66	860.7005	1	305.5	0.14
GlcCer 46:05 OH*	[M+H]	309.9	-0.69	876.6957	1	307.8	0.14
	**Average absolute % bias**		**0.43**			**Average % RSD**	**0.14**
**Sphingomyelins**							
SM 34:01*	[M+H]	281.2	0.18	703.5808	1	281.7	0.13
SM 34:01	[M+Na]	279.9	0.87	725.5647	1	282.3	0.22
SM 36:01	[M+H]	288.4	-0.36	731.6116	1	287.4	0.15
SM 36:01*	[M+Na]	284.1	1.32	753.5268	1	287.9	0.15
SM 36:02	[M+H]	285.3	-0.51	729.5966	1	283.9	0.15
SM 38:00	[M+H]	289.8	0.16	745.6293	1	290.3	0.18

SM 38:01	[M+H]	293.4	-0.11	759.6422	1	293.1	0.15
SM 38:01	[M+Na]	293.3	0.04	781.6248	1	293.4	0.12
SM 40:00*	[M+H]	300.8	0.08	789.6903	1	301.0	0.14
SM 40:01	[M+H]	299.1	-0.10	787.6743	1	298.8	0.14
SM 40:01	[M+Na]	297.2	0.62	809.6581	1	299.0	0.13
SM 40:02*	[M+H]	296.8	-0.31	785.6599	1	295.9	0.15
SM 41:01*	[M+H]	302.3	-0.18	801.6911	1	301.7	0.15
SM 41:02	[M+H]	300.1	-0.49	799.6761	1	298.6	0.19
SM 42:00	[M+H]	306.5	0.11	817.7225	1	306.8	0.14
SM 42:01	[M+H]	304.4	-0.05	815.7058	1	304.3	0.14
SM 42:02	[M+H]	302.2	-0.19	813.6896	1	301.6	0.15
SM 42:02	[M+Na]	302.0	-0.28	835.6737	1	301.2	0.14
SM 42:03	[M+H]	300.8	-0.66	811.6745	1	298.8	0.19
SM 44:01	[M+H]	305.7	-0.29	827.7070	1	304.8	0.17
SM 44:02	[M+H]	311.0	-0.39	843.7410	1	309.8	0.21
SM 44:03*	[M+H]	308.7	-0.34	841.7226	1	307.6	0.14
SM (*m/z* 733.63)	--	--	--	733.6272	1	290.1	0.13
SM (*m/z* 761.66)	--	--	--	761.6590	1	295.6	0.14
	**Average absolute % bias**		**0.35**		**Average % RSD**		**0.15**
**SPLASH Mix**							
18:1(d7) LPC	[M+H]	232.9	-0.53	529.4026	1	231.7	0.09
18:1(d7) LPC	[M+Na]	236.0	-1.14	551.3809	1	233.4	0.10
15:0-18:1(d7) DG	[M+H-H2O]	255.6	0.75	570.5576	1	256.7	0.16
15:0-18:1(d7) DG	[M+Na]	257.1	-0.18	610.5485	1	257.6	0.12
15:0-18:1(d7) PE	[M+H]	271.7	0.31	711.5758	1	272.6	0.10
15:0-18:1(d7) PE	[M+Na]	277.8	0.03	733.5595	1	277.9	0.11
15:0-18:1(d7) PS	[M+H]	278.6	0.23	755.5674	1	279.2	0.10
18:1(d9) SM	[M+H]	286.3	0.71	738.6596	1	287.5	0.10
18:1(d9) SM	[M+Na]	286.9	0.22	760.6386	1	288.4	0.11
15:0-18:1(d7)-15:0 TG	[M+NH4]	312.1	-0.11	829.8109	1	309.3	0.10
15:0-18:1(d7)-15:0 TG	[M+Na]	310.6	-0.41	834.7644	1	311.7	0.10
	**Average absolute % bias**		**0.42**		**Average % RSD**		**0.11**

Calibration Simulation for Evaluation of Calibrant Error Contribution

A calibration simulation was performed to assess the contribution of uncertainty associated with the reference HFAP ^{DT}CCS values. For each HFAP ion, 10 CCS values were selected from a uniform distribution centered around the accepted value with the width of the distribution corresponding to the uncertainty of the measurement, reported as %RSD (**Table S3**). The resulting values are tabulated below (**Table S4**).

Table S3. HFAP calibrant CCS values.

HFAP ion	Arrival time (ms)	^{DT}CCS	RSD
m/z 622	223.66	202.8	0.12%
m/z 922	349.34	243.5	0.11%
m/z 1222	487.28	281.8	0.10%
m/z 1522	629.68	316.5	0.10%
m/z 1822	782.37	351.2	0.18%
m/z 2122	941.26	383.0	0.17%
m/z 2422	1103.69	412.9	0.14%
m/z 2722	1270.02	441.2	0.13%

Table S4. HFAP simulated CCS values.

HFAP ion	CCS (1)	CCS (2)	CCS (3)	CCS (4)	CCS (5)	CCS (6)	CCS (7)	CCS (8)	CCS (9)	CCS (10)
m/z 622	202.63	202.70	202.81	203.00	202.65	203.01	202.60	203.02	202.68	202.85
m/z 922	243.76	243.40	243.41	243.58	243.65	243.40	243.38	243.62	243.37	243.27
m/z 1222	281.77	281.68	281.99	282.06	281.78	281.73	282.07	281.64	281.65	281.78
m/z 1522	316.68	316.40	316.48	316.59	316.36	316.35	316.79	316.25	316.24	316.33
m/z 1822	351.64	351.48	351.22	351.26	351.19	351.56	350.67	351.48	351.79	350.83
m/z 2122	383.10	382.95	382.66	383.28	382.87	383.32	382.59	382.69	382.96	383.25
m/z 2422	413.26	412.84	413.08	413.21	413.11	413.38	412.39	413.31	412.34	412.38
m/z 2722	440.90	441.35	441.49	441.08	441.72	441.30	440.94	440.95	440.87	441.10

For each of the ten sets of CCS values, a calibration was performed using a trinomial equation. This resulted in the coefficients listed in **Table S5**.

Table S5. Calibration coefficients for simulated calibrations.

Coeff.	(1)	(2)	(3)	(4)	(5)	(6)	(7)	(8)	(9)	(10)
A	2.38E-07	2.52E-07	2.67E-07	2.36E-07	2.66E-07	2.18E-07	2.86E-07	2.27E-07	2.44E-07	2.47E-07
B	-9.67E-04	-9.89E-04	-1.02E-03	-9.55E-04	-1.02E-03	-9.14E-04	-1.07E-03	-9.31E-04	-9.79E-04	-9.78E-04
C	2.20E+00	2.20E+00	2.22E+00	2.18E+00	2.22E+00	2.16E+00	2.25E+00	2.16E+00	2.20E+00	2.19E+00
D	6.06E+02	6.05E+02	6.03E+02	6.10E+02	6.04E+02	6.14E+02	5.97E+02	6.14E+02	6.05E+02	6.08E+02

Applying these calibration equations to the PC lipid extract data resulted in an average uncertainty in the calibrated CCS values of 0.04% (**Table S6**).

Table S6. Summary of calibrated CCS values of PC lipids using the simulated calibrant values.

Compound	Arrival time (ms)	Avg. calibrated CCS	Standard deviation	RSD
PC 34:03	499.31	286.8	0.1159	0.040%
PC 34:02	509.89	289.6	0.1158	0.040%
PC 34:01	521.66	292.8	0.1155	0.039%
PC 34:01	526.44	294.0	0.1153	0.039%
PC 34:02	518.68	291.8	0.1156	0.040%
PC 34:01	531.21	295.1	0.1150	0.039%
PC 36:02	536.05	296.4	0.1147	0.039%
PC 36:03	532.08	295.2	0.1149	0.039%
PC 36:01	551.85	300.5	0.1138	0.038%
PC 36:01	546.60	299.1	0.1141	0.038%
PC 36:02	543.39	298.2	0.1142	0.038%
PC 36:01	556.83	301.6	0.1134	0.038%
PC 36:01	547.63	299.2	0.1140	0.038%
PC 38:03	558.81	302.0	0.1132	0.038%
